# Treatment of chronic plantar fasciopathy with extracorporeal shock waves (review)

**DOI:** 10.1186/1749-799X-8-31

**Published:** 2013-09-03

**Authors:** Christoph Schmitz, Nikolaus BM Császár, Jan-Dirk Rompe, Humberto Chaves, John P Furia

**Affiliations:** 1 Department of Anatomy II, Ludwig-Maximilians-University of Munich, Pettenkoferstr. 11, Munich, 80336, Germany; 2 OrthoTrauma Evaluation Center, Oppenheimer Str. 70, Mainz, 55130, Germany; 3 Institute of Mechanics and Fluid Dynamics, TU Bergakademie Freiberg, Lampadiusstr. 4, Freiberg, 09596, Germany; 4 Sun Orthopaedic Group, Inc., 900 Buffalo Rd., Lewisburg, PA, 17837, USA

**Keywords:** Extracorporeal shock wave treatment (ESWT), Radial extracorporeal shock wave treatment (RSWT), Plantar fasciitis, Plantar fasciopathy

## Abstract

There is an increasing interest by doctors and patients in extracorporeal shock wave therapy (ESWT) for chronic plantar fasciopathy (PF), particularly in second generation radial extracorporeal shock wave therapy (RSWT). The present review aims at serving this interest by providing a comprehensive overview on physical and medical definitions of shock waves and a detailed assessment of the quality and significance of the randomized clinical trials published on ESWT and RSWT as it is used to treat chronic PF. Both ESWT and RSWT are safe, effective, and technically easy treatments for chronic PF. The main advantages of RSWT over ESWT are the lack of need for any anesthesia during the treatment and the demonstrated long-term treatment success (demonstrated at both 6 and 12 months after the first treatment using RSWT, compared to follow-up intervals of no more than 12 weeks after the first treatment using ESWT). In recent years, a greater understanding of the clinical outcomes in ESWT and RSWT for chronic PF has arisen in relationship not only in the design of studies, but also in procedure, energy level, and shock wave propagation. Either procedure should be considered for patients 18 years of age or older with chronic PF prior to surgical intervention.

## Introduction

Plantar fasciitis (PF), the most common cause of heel pain, accounts for approximately 11% to 15% of foot symptoms presenting to physicians. In the United States, more than 2 million individuals are treated for PF on an annual basis [1-3]. The term ‘plantar fasciitis’ implies an inflammatory condition by the suffix ‘itis’. However, various lines of evidence indicate that this disorder is better classified as ‘fasciosis’ or ‘fasciopathy’, as heel pain is associated with degenerative changes in the fascia and atrophy of the abductor minimi muscle [1]. When chronic, PF is not an inflammatory condition [1].

The etiology, pathogenesis, associated risk factors, and general treatment strategies for PF have been documented in other comprehensive reviews [1-6]. The condition is usually diagnosed clinically based on the history of morning heel pain made worse with ambulation on hard surfaces and by the physical findings of pain over the medial aspect of the plantar fascia. PF has a bimodal distribution, afflicting both athletes and the sedentary. Imaging studies, while generally not needed, can be helpful for ruling out other causes of heel pain or to establish the diagnosis of PF when in doubt.

Initial treatment is non-operative and consists of relative rest, physical therapy, stretching, exercises, shoe inserts/orthotics, night splints, non-steroidal anti-inflammatory drugs, and local corticosteroid injections. Patients not responding to conservative treatment for 4 to 6 months (between 10% and 20% of all patients) are candidates for more aggressive treatment such as extracorporeal shock wave therapy (ESWT) and surgery [1,2].

The safety and efficacy of ESWT for chronic PF has been assessed in a variety of randomized clinical trials (RCTs). Rompe et al. [5] have already reviewed the results of using focused shock wave therapy to treat chronic PF. Since then, five RCTs have assessed the safety and efficacy of radial extracorporeal shock wave therapy (RSWT) for chronic PF [7-11]. Recently, Dizon et al. [12] reviewed the results of using both ESWT and RSWT for chronic PF [12].

The following review provides an overview on the physical and medical definitions of shock waves, as well as a detailed assessment of the quality and significance of the recently published RCTs on both ESWT and RSWT for chronic PF.

### Shock waves

#### Physical definition of shock waves

A shock wave is an acoustic pressure wave that is produced in any elastic medium such as air, water, or even a solid substance [13,14]. Shock waves differ from sound waves in that the wave front, where compression takes place, is a region of sudden change in stress and density [13,14]. Because of this, shock waves propagate in a manner different from that of the ordinary acoustic waves. In particular, shock waves travel faster than sound, and their speed increases as the amplitude is raised; however, the intensity of a shock wave also decreases faster than does that of a sound wave because some of the energy of the shock wave is expended to heat the medium in which it travels [13,14].

**Figure 1:**
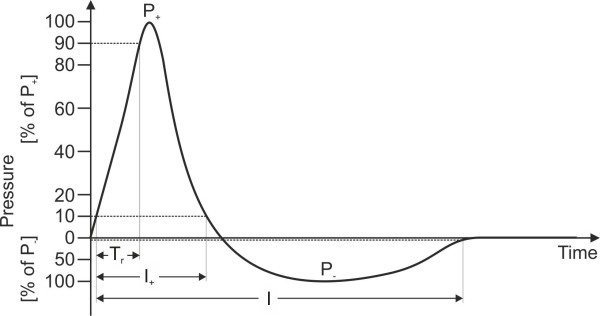
**Pressure as a function of time of a shock wave.** P_+_, positive peak pressure; P_−_, negative peak pressure; T_r_, rise time (i.e., the time interval during which the positive pressure changes from 10% of P_+_ to 90% of P_+_); I_+_, time interval used to calculate the positive energy of the shock wave; I, time interval to calculate the total energy of the shock wave.

Accordingly, shock waves are characterized by (1) a high positive peak pressure (P_+_), sometimes more than 100 MPa but more often approximately 50 to 80 MPa, (2) a fast initial rise in pressure (T_r_) during a period of less than 10 ns, (3) a low tensile amplitude (P_−_, up to 10 MPa), (4) a short life cycle (I) of approximately 10 μs, and (5) a broad frequency spectrum, typically in the range of 16 Hz to 20 MHz [15,16]. The measured shock wave rise time is in the 30-ns range when determined by limited time resolution of the pressure recording hydrophone [15,16]. The positive pressure amplitude is followed by a diffraction-induced tensile wave of a few microseconds duration (Figure 1).

### Medical definitions of shock waves

Extracorporeal shock wave lithotripsy (ESWL) is widely used for stone management in urology. The pressure waves applied in stone management fulfill the characteristics set out by the physical definition of shock waves provided above [17]. The fast initial rise time in pressure and the high positive pressure causes a pressure gradient within the renal calculi that, when of sufficient energy, can fragment the calculi [18].

ESWT and RSWT are by-products of lithotriptor technology. They were introduced into the treatment for various diseases of the musculoskeletal system, such as PF, Achilles tendinopathy, medial tibial stress syndrome, greater trochanteric pain syndrome, lateral and medial epicondylitis, and calcifying tendonitis of the shoulder since the late 1980s [19].

Shock waves have both a direct and indirect effect on treated tissues. The direct effect is the result of the energy of the shock wave being transferred to the targeted tissues. The indirect effect is the result of the production of cavitation bubbles in the treated tissue [13,14]. Both the direct and indirect effects produce a biological response in the treated tissues.

### Shock wave generators

In the United States, the following ESWT/RSWT devices obtained premarket approval (PMA) by the Food and Drug Administration (FDA) as class III orthopedic lithotripsy devices and were reclassified as class III Generators, Shock Wave, For Pain Relief (Product Code NBN) in the Spring 2009: (1) Ossatron (HealthTronics, Inc., Marietta, GA, USA), PMA # P990086 issued on October 12, 2000 to treat chronic heel pain; (2) Epos Ultra (Dornier Medical Systems, Inc., Kennesaw, GA, USA), PMA # P000048 issued on January 15, 2002 for the treatment of chronic plantar fasciitis for patients with symptoms of plantar fasciitis for 6 months or more and a history of unsuccessful conservative therapy; (3) Sonocur Basic (Siemens Medical Solutions, Inc., Iselin, NJ, USA), PMA # P010039 issued on July 19, 2002 for the treatment for pain due to tennis elbow; (4) Orthospec Extracorporeal Shock Wave Therapy (Medispec, Ltd; Germantown, MD, USA), PMA # P040026 issued on April 1, 2005 for the treatment of proximal plantar fasciitis with or without heel spur in patients 18 years of age or older; (5) Orbasone Pain Relief System (Orthometrix, Inc., White Plains, NY, USA), PMA # P040039 issued on August 10, 2005 to relieve heel pain (proximal plantar fasciitis); and (6) Swiss DolorClast (EMS Electro Medical Systems; Dallas, TX, USA), PMA # P050004 issued on May 8, 2007 to treat heel pain associated with chronic proximal plantar fasciitis [20].

The Ossatron, Epos Ultra, Sonocur Basic, and Orbasone devices share the following technical key characteristics of ESWL devices used for stone management: (1) electrohydraulic (OssaTron, Orbasone) or electromagnetic (Epos Ultra, Sonocur) generation of pressure waves and (2) generation of focused pressure waves. The Orthospec device also uses electrohydraulic spark gap technology to generate pressure waves. The Swiss DolorClast generates radial pressure waves ballistically, i.e., by accelerating a bullet to strike an applicator, which transforms the kinetic energy of the bullet into a radially expanding pressure wave [8,21].

It should be noted that studies by Chitnis and Cleveland [22] and Cleveland et al. [23] showed that the Swiss DolorClast does not generate pressure waves that fulfill the characteristics set out by the physical definition of shock waves provided above. Specifically, the rise time of the pressure waves generated by the Swiss DolorClast was reported as 600 [22] or 800 ns [23], respectively. This rise time is approximately 90 times longer than would be expected for a shock wave [23]. Furthermore, the maximum peak positive pressure of the Swiss DolorClast was reported as 5 [22] or 7 MPa [23], respectively. Accordingly, the question has been raised whether the pressure waves generated with the Ossatron, Epos Ultra, Sonocur Basic, Orbasone, and Orthospec devices fulfill the characteristics set out by the physical definition of shock waves provided above. This is addressed in the Appendix.

### Treatment of chronic plantar fasciopathy with focused shock waves

Rompe et al. [5] assessed the quality of all RCTs on focused ESWT for chronic PF that were published in the international peer-reviewed literature until then. To this end, the authors applied evaluation criteria established by Chalmers et al. [24], consisting of two evaluation forms that include 29 individually scored items, allowing a maximum score of 100. Besides this, Rompe et al. [5] used evaluation criteria established by Jadad et al. [25], attributing to each RCT a quality score out of a maximum of six points, addressing the following questions: (1) was the generation of randomization sequence described? (2) was the method of allocation concealment described? (3) was an intention to treat analysis used? (4) what number of patients was lost to follow up? (5) was the outcome assessment blind? and (6) was the patient blind to treatment allocation?

This assessment resulted in the following quality scores (see also Table 1): (1) Haake et al. [26], 90 (according to Chalmers et al. [24])/6 (according to Jadad et al. [25]); (2) Kudo et al. [27], 88/6; (3) Malay et al. [28], 84/6; and (4) Buchbinder et al. [29], 82/4. All other RCTs had lower scores and were not classified as *good* by Rompe et al. [5]; the same holds true for the only RCT on focused ESWT for chronic PF published since then by Gollwitzer et al. [30]. Dizon et al. [12] assessed the same studies using the so-called physiotherapy evidence database (PEDro) scale [31] and ranked them as follows (maximum scale = 11): (1) Kudo et al. [27], Malay et al. [28], and Buchbinder et al. [29], 10; (2) Gollwitzer et al. [30], 9; and (3) Haake et al. [26], 8. Because of the difference between the assessments by Rompe et al. [5] and Dizon et al. [12] and considering that Rompe et al.’s [5] assessment criteria are more sensitive than the assessment criteria used by Dizon et al. [12], the latter was no longer considered in this review.

**Table 1:** **RCTs on ESWT for chronic PF classified as *good *by Rompe et al. [**[5]**]**

**Study**	**Outcome**	**Chalmers score**^**c**^	**Jadad score**^**d**^	**Placebo treatment**	**L.A.**	**Patients**
Haake et al. [26]	−^a^	90	6	^e^	+^h^	^j^
Kudo et al. [27]	+^b^	88	6	^e^	−^i^	^k^
Malay et al. [28]	+^b^	84	6	^f^	−	^k^
Buchbinder et al. [29]	−^a^	82	4	^g^	−	^j^

^a^Lack of statistically significantly (*p* < 0.05) better outcome for the patients treated with ESWT than the patients treated with placebo; ^b^statistically significantly (*p* < 0.05) better outcome for the patients treated with ESWT than the patients treated with placebo; ^c^quality score according to Chalmers et al. [24]; ^d^quality score according to Jadad et al. [25]; ^e^treatment of patients in the placebo group in exactly the same manner as patients in the active group but placing a foam-insulated or air-filled membrane between the applicator of the shock wave device and the patient’s skin that reflects the shock waves; ^f^treatment of patients in the placebo group in exactly the same manner as patients in the active group but modifying the shock wave device so that it does not deliver shock waves; ^g^treatment of patients in the placebo group with only a small number of shock waves at low energy settings; ^h^local anesthesia; ^i^medial calcaneal nerve block anesthesia; ^j^patients with symptoms present for less or more than 6 months who have or have not previously failed pharmacologic (analgesic, anti-inflammatory, or other) and non-pharmacologic treatment modalities for the relief of heel pain; ^k^only patients with symptoms present for more than 6 months who have previously failed pharmacologic (analgesic, anti-inflammatory, or other) and non-pharmacologic treatment modalities for relief of heel pain.

Interestingly, Kudo et al. [27] and Malay et al. [28] found statistically significantly (*p* < 0.05) better outcomes for the patients treated with ESWT than the patients treated with placebo whereas Haake et al. [26] and Buchbinder et al. [29] did not. At first glance, this may be confusing as Haake et al. [26], Kudo et al. [27], and Buchbinder et al. [29] used the same ESWT device (namely, the Epos Ultra). As outlined in the following, however, this discrepancy can be explained by methodological differences between these studies, going beyond the generalized quality criteria for RCTs established by Chalmers et al. [24], Jadad et al. [25], or the PEDro scale [31]. Specifically, application of placebo treatment, patient blinding, and the use of local anesthesia in RCTs with ESWT for chronic PF needs to be addressed (Table 1).

### Application of placebo treatment and patient blinding

The application of placebo treatment in RCTs with ESWT for chronic PF can be achieved in three different ways: (1) treating patients in the placebo group in exactly the same manner as patients in the active group but modifying the shock wave device so that it does not deliver shock waves. This was done in the study by Malay et al. [28] using a foam-insulated contact membrane placed on the applicator that absorbed the shock waves and inhibited transmission of most of the energy but looked identical to an unlined (non-insulated) contact membrane placed on the applicator when treating patients in the active group. (2) Treating patients in the placebo group in exactly the same manner as patients in the active group but placing a foam-insulated or air-filled membrane between the applicator of the shock wave device and the patient’s skin that reflects the shock waves. This was done in the studies by Haake et al. [26] and Kudo et al. [27]. (3) Treating patients in the placebo group with only a small number of shock waves at low energy settings. This was applied in the study by Buchbinder et al. [29]. Specifically, Buchbinder et al. [29] treated patients in the placebo group with only 100 impulses with energy flux density (EFD) of 0.02 mJ/mm^2^ per treatment session, but patients in the active group were treated with 2,000 or 2,500 impulses with EDF varying between 0.02 and 0.33 mJ/mm^2^ per treatment session.

From a methodological point of view, the first option provides the best patient blinding and is the only one in which unblinding of the patients by the ESW therapist can be excluded (provided that the preparation of the device is performed by an independent person, or the ESW therapist is provided with coded ‘active’ and ‘placebo’ handpieces; note that in the study by Malay et al. [28], the ESW therapists were not blinded). The second and third options do not keep the ESW therapists ‘unaware’ of the assigned treatment, opening the possibility that they could be influenced by that knowledge. This requires a strict, standardized way of interaction between the ESW therapist and the patients, irrespective of the treatment allocation (as mentioned in the study by Buchbinder et al. [29]). Moreover, the third option inherits the highest chance of patients’ unblinding because patients could conclude from the knowledge that is available freely that they were not in the active group. For example, treatment of chronic PF with focused ESWT devices can last for more than 10 min (because the frequency to apply the shock waves using these devices is limited to a few Hz) and can be very painful for the patients if applied without anesthesia (as performed in the study by Buchbinder et al. [29]). However, in the study by Buchbinder et al. [29], the placebo treatment lasted less than 2 min (only 100 impulses applied at a frequency of 1 Hz), whereas the active treatment lasted more than 10 min (2,000 or 2,500 impulses applied at frequencies that were gradually increased to 4 Hz). Thus, in the study by Buchbinder et al. [29], the patients in the placebo group received a painless treatment during a short treatment time, whereas the patients in the active group received a painful treatment during a substantially longer treatment time (note that the active treatment was actually gradually increased through to the highest tolerable level of pain for each participant in this study).

Accordingly, the quality of patient blinding (and thus allocation concealment) in the aforementioned studies can be ranked as follows: Malay et al. [28] > Haake et al. [26] = Kudo et al. [27] > Buchbinder et al. [29].

### Use of local anesthesia

The pain associated with the application of focused shock waves and the need for patient blinding in RCTs testing painful treatment modalities imply that RCTs with ESWT for chronic PF should be performed under local anesthesia (as done by Haake et al. [26]) or nerve block anesthesia (as done by Kudo et al. [27]). However, the application of local anesthesia might contribute to negative outcome of such studies, as demonstrated by Rompe et al. [32]. The molecular mechanisms underlying this phenomenon are not yet fully understood, but substantial evidence points to a central role of the peripheral nervous system in mediating molecular and cellular effects of shock waves applied to the musculoskeletal system [33-35]. These effects could be blocked by local anesthesia, as demonstrated in a recent study by Klonschinski et al. [36]. Thus, it is now generally recommended to apply shock waves without local anesthesia to the musculoskeletal system.

Kudo et al. [27] did not use local anesthesia, but a medial calcaneal nerve block anesthesia. The authors applied a total of 3,800 impulses in a single session with an average EFD of 0.34 mJ/mm^2^. In contrast, Malay et al. [28] did not use any anesthesia. These authors applied a total of 3,800 impulses in a single session, divided into approximately 540 impulses for each of the seven energy levels of the device used (Orthospec) (resulting in an average EFD of 0.2 mJ/mm^2^). Buchbinder et al. [29] did not use any anesthesia either. The average EFD per impulse in this study (2,000 or 2,500 impulses per treatment session, three treatment sessions, average total EFD = 1,406.73 mJ/mm^2^) was approximately 0.21 mJ/mm^2^.

Accordingly, with respect to the use of local anesthesia, the aforementioned studies can be ranked as follows: Malay et al. [28] = Buchbinder et al. [29] > Kudo et al. [27] > Haake et al. [26].

### Significance of published, randomized clinical trials on focused shock wave treatment for chronic plantar fasciopathy

Considering the aforementioned methodological issues, the RCTs using focused ESWT for chronic PF that were evaluated as good by Rompe et al. [5] can be assessed as follows.

Buchbinder et al. [29] included a total of *n* = 166 patients with symptoms of PF for at least 6 weeks (range 8 to 980 weeks) into their study. Patients in the active group received three sessions of ESWT at weekly intervals, with 2,000 or 2,500 impulses with EFD varying between 0.02 mJ/mm^2^ and 0.33 mJ/mm^2^ per treatment session. Patients in the placebo group received only 100 impulses with EFD = 0.02 mJ/mm^2^ per treatment session.

At 6 and 12 weeks, there were significant improvements in overall pain in both the active group and the placebo group. Similar improvements in both groups were also observed for morning and activity pain, walking ability, and other end points. However, there were no statistically significant (*p* < 0.05) differences in the degree of improvement between the groups for any measured outcomes.

It is important to recognize that Buchbinder et al. [29] did not treat chronic PF according to the classic definition of ‘chronic’ (i.e., patients not responding to conservative treatment for 6 months). Rather, Buchbinder et al. [29] investigated mixed groups of patients suffering from either acute (as short as 8 weeks) or chronic PF. Furthermore, it appears that not all of the patients enrolled by Buchbinder et al. [29] received conservative care before inclusion in the study. For example, only 54% (90/166) of the patients were treated with orthotics before ESWT, only 31% (51/166) received cortisone injections before ESWT, and only 13% (21/166) were treated with physiotherapy before ESWT.

Haake et al. [26] treated a total of *n* = 272 patients with three sessions of 4,000 focused shock waves with EFD = 0.08 mJ/mm^2^ under local anesthesia or placebo at weekly intervals. After 12 weeks, the success rate was 34% in the ESWT group and 30% in the placebo group; the difference was not statistically significant (*p* > 0.05).

This study was criticized because fewer than half of the enrolled patients received minimal conservative care such as stretching exercises, casting, or night splinting before inclusion in the study (similar to the situation in the study by Buchbinder et al. [29]) [37]. Furthermore, the lack of treatment success in the ESWT group in the study by Haake et al. [26] can be explained by the fact that these authors applied shock waves under local anesthesia.

Kudo et al. [27] in a trial of 114 patients with chronic PF, recalcitrant to conservative therapies for at least six months, achieved treatment success by applying focused shock waves (single sessions of 3,800 impulses with EFD = 0.34 mJ/mm^2^) or placebo treatment under medial calcaneal nerve block anesthesia. Good or excellent outcome was reported by 43% of the patients treated with focused shock waves at 12-weeks follow-up and by 30% of the placebo-treated patients (*p* < 0.05). Kudo et al. [27] did not report the results at longer than 12 weeks after the treatment.

Malay et al. [28] included a total of *n* = 172 patients with symptoms present for more than 6 months into their study. Patients must have previously failed two pharmacologic (analgesic, anti-inflammatory, or other) and two non-pharmacologic treatment modalities for the relief of heel pain to be included in the study. Patients were treated with a single session of 3,800 shock waves or placebo, without local anesthesia. The energy flux density of the applied shock waves was continuously increased from 0.08 mJ/mm^2^ (lowest energy level of the used device) to 0.33 mJ/mm^2^ (highest energy level of the used device). At 12 weeks, 43% of the patients treated with shock waves and 20% of the patients treated with placebo reported a 50% decrease of pain from baseline (statistically significant, *p* < 0.05). As with Kudo et al.’s [27] trial, Malay et al. [28] did not follow up with the patients longer than 12 weeks after the treatment.

In summary, the lack of treatment success in the studies by Buchbinder et al. [29] and Haake et al. [26] using focused ESWT for PF can be explained by serious methodological shortcomings in the corresponding studies. In contrast, the studies by Kudo et al. [27] and Malay et al. [28] demonstrated that chronic PF can be treated successfully with focused shock waves. Furthermore, the study by Malay et al. [28] showed that treatment success can be achieved without any anesthesia. However, long-term (>12 weeks) treatment success has not been demonstrated in either of these trials.

### Treatment of chronic plantar fasciopathy with radial shock waves

Five RCTs have assessed the safety and efficacy of RSWT for chronic PF [7-11]. Two of these studies (Gerdesmeyer et al. [8] and Ibrahim et al. [11]) fulfilled all evaluation criteria established by Chalmers et al. [24] and Jadad et al. [25] outlined above. Using the PEDro scale [31], Dizon et al. [12] ranked these studies as follows: (1) Gerdesmeyer et al. [8] and Ibrahim et al. [11], 10; (2) Chow and Cheing [7], 9; and (3) Greve et al. [10], 7. The study by Marks et al. [9] was not considered by Dizon et al. [12]. Furthermore, according to Dizon et al. [12], the therapists were not blinded in the study by Gerdesmeyer et al. [8]. However, this is not correct. Gerdesmeyer et al.’s [8] study was a double-blind, randomized, placebo-controlled trial and, thus, should have received a PEDro scale score of 11 in Dizon et al.’s [12] assessment.

**Figure 2:**
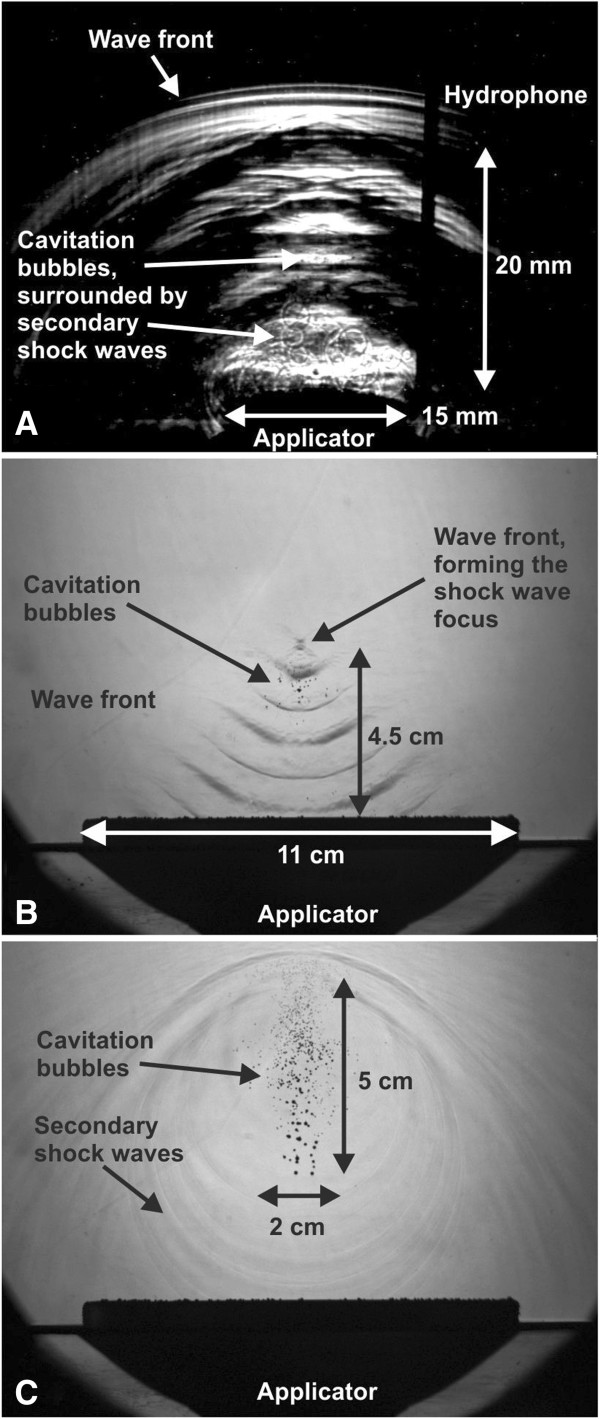
Shadowgraph images of radial (A) and focused (B)shock waves (details are provided in the main text).

Radial shock waves can be delivered to the tissue without local or nerve block anesthesia, and no form of anesthesia was used in the aforementioned trials. In general, radial extracorporeal shock wave therapy is better tolerated than focused SWT because radial shock waves have their point of highest pressure and highest energy flux density at the tip of the applicator and, thus, outside the tissue. In contrast, focused shock waves have their point of highest pressure and highest energy flux density at the center of their focus which is positioned within the treated tissue. This is demonstrated in Figure 2 showing shadowgraph images of radial and focused shock waves used for the treatment of the musculoskeletal system. Shadowgraph imaging is a visual process that is used to photograph the flow of fluids of varying density. Figure 2A shows radial shock waves generated with the Swiss DolorClast. Note the semicircular wave front and the field of cavitation bubbles surrounded by secondary shock waves above the applicator. The secondary shock waves are produced by rapid collapse of the cavitation bubbles (this process is named inertial cavitation). The cavitation is consequent to the negative phase of the wave propagation. The cavitation field produced with the Swiss DolorClast (15-mm applicator, device operated at 4 bar) has a size of approximately 15 × 20 mm (width × height). The hydrophone is used to trigger the flash and the camera. Figure 2B shows shock waves generated with a focused shock wave device, Swiss Piezoclast (Electro Medical Systems; the Swiss Piezoclast is currently not FDA approved). Note the convergent waves and the center of the shock wave focus at a height of 4.5 cm above the applicator. Cavitation bubbles appear near the center of the shock wave focus. The picture was generated by mounting five shadowgraph images taken each at 12 μs apart into one figure. The device was operated so that the shock waves had a rise time of 79 ns and a positive peak pressure of 82.8 MPa. Figure 2C shows the cavitation field of the Swiss Piezoclast surrounded by secondary shock waves, observed at 31.6 μs after forming the center of the shock wave focus. The cavitation field has an elliptic shape with equatorial diameter of approximately 2 cm and polar diameter of approximately 5 cm. Note that the so-called 5-MPa focus (i.e., the region in which the positive pressure exceeds 5 MPa during the positive phase of the wave propagation) of the shock waves generated with the Swiss Piezoclast has an equatorial diameter of 20.8 mm, which equals the equatorial diameter of the cavitation field caused by the shock waves generated with the Swiss Piezoclast. It is of note that the size of the cavitation field depends on the medium in which the shadowgraph images are taken. The images shown here were taken in non-degassed water. In contrast, Chitnis and Cleveland [22] investigated the cavitation fields of the Ossatron and Swiss DolorClast devices in degassed water and obtained the following results: Ossatron, 10 × 40 mm (width × height) and Swiss DolorClast, 3 × 3 mm (i.e., five times smaller in width and height as demonstrated here). Assuming linear relationships between the results of Chitnis and Cleveland [22] and the findings presented here, one would expect that the cavitation field of the Ossatron device has an equatorial diameter of approximately 50 mm when evaluated in non-degassed water. Interestingly, the 5-MPa focus of the shock waves generated with the Ossatron device has an equatorial diameter of 64 mm, which equals the equatorial diameter of the assumed cavitation field caused by the shock waves generated with this device in non-degassed water.

Thus, radial shock waves of a certain energy flux density are generally less painful for and, thus, better tolerated by the patient than the focused shock waves of the same energy flux density.

Gerdesmeyer et al. [8] demonstrated safety and efficacy of RSWT with the Swiss DolorClast for chronic PF in a prospective, randomized, double-blinded, placebo-controlled international multicenter study. The authors included a total of *n* = 245 patients with chronic PF into their study. Inclusion criteria comprised (among others) a history of at least 6 months of chronic plantar painful heel syndrome that proved resistant to non-surgical treatment. Gerdesmeyer et al. [8] administered RSWT or placebo treatment in three sessions, each at 2 weeks (±4 days) apart (2,000 impulses per session, EFD = 0.16 mJ/mm^2^, eight impulses per second) and evaluated the treatment outcome 12 weeks and 12 months after the first session. The authors found a statistically significant (*p* < 0.05) difference in the reduction of the mean visual analog scale (VAS) composite score between the patients treated with RSWT (−56.0% ± 39.3%) and the placebo-treated patients (−44.1% ± 41.8%) at 12 weeks and even more pronounced superiority of RSWT (−61.9% ± 43.6%) over placebo (−46.5% ± 45.5%) at 12 months.

Ibrahim et al. [11] tested (in a prospective, randomized, double-blinded, placebo-controlled study) the hypothesis that treatment of chronic PF with two RSWT sessions 1 week apart does result in profound pain relief compared to placebo treatment 4 weeks after the first RSWT treatment, lasting for at least 6 months. To test this hypothesis, the authors randomly assigned a total of *n* = 50 patients with unilateral, chronic PF to either RSWT (*n* = 25) or placebo treatment (*n* = 25). Inclusion and exclusion criteria were almost identical to those applied by Gerdesmeyer et al. [8]. RSWT was applied in two sessions 1 week apart (2,000 impulses with EFD = 0.16 mJ/mm^2^ per session). Placebo treatment was performed with a clasp on the heel. End points were changes in the VAS score and the modified Roles and Maudsley (RM) score from baseline to 4-, 12-, and 24-week follow-up. Ibrahim et al. [11] found the mean VAS scores reduced after RSWT from 8.52 ± 0.34 (mean ± SEM) at baseline to 0.64 ± 1.52 at 4 weeks, 1.08 ± 0.28 at 12 weeks, and 0.52 ± 0.14 at 24 weeks from baseline. Similar changes were found for mean RM scores after RSWT but were not observed after placebo treatment. Statistical analysis demonstrated that RSWT resulted in significantly reduced mean VAS scores and mean RM scores at all follow-up intervals compared to placebo treatment (each with *p* < 0.001). No serious adverse events of RSWT were observed. Ibrahim et al. [11] concluded that RSWT is efficient in the treatment for chronic PF even when only two sessions with 2,000 impulses each are performed 1 week apart.

To investigate the dose-effect relationship of RSWT to treatment success, Chow and Cheing [7] randomly assigned a total of *n* = 57 patients with chronic PF for at least 3 months to three groups. Patients in group A (*n* = 19, 17 patients completed the trial) received three sessions of RSWT each 1 week apart (1,000 impulses per session, EFD = 0.11 mJ/mm^2^, three impulses per second). Patients in group B (*n* = 19, 18 patients completed the trial) were treated in the same manner but with increasing energy flux densities (first week, EFD = 0.12 mJ/mm^2^; second week, EFD = 0.15 mJ/mm^2^; third week, EFD = 0.17 mJ/mm^2^). Patients in group C served as control (*n* = 19, 14 patients completed the trial; three sessions of RSWT each 1 week apart, 30 impulses per session, EFD = 0.03 mJ/mm^2^, three impulses per second). Six weeks after the first RSWT session, patients in groups A and B showed (among other variables) statistically significant (*p* < 0.05) reductions in mean VAS scores by 37% (group A) and 83% (group B), respectively, compared to the baseline. By contrast, patients in group C showed no changes in mean VAS scores compared to the baseline. The results of the patients in group B of the study by Chow and Cheing [7] were consistent with the results reported by Gerdesmeyer et al. [8] and Ibrahim et al. [11], indicating that the energy flux density of the applied radial shock waves must exceed a certain level in order to cause a therapeutic effect.

Other trials investigating the use of RSWT to treat chronic PF have yielded negative outcomes. Marks et al. [9] enrolled 25 adult patients with chronic PF in their study. The authors randomly assigned 16 patients to RSWT (three sessions each 3 days apart, 500 impulses in the first session and 2,000 impulses in the second and third session, respectively; EFD = 0.16 mJ/mm^2^, frequency of the impulses not provided). Another nine patients were placebo treated (i.e., in the same manner as the patients subjected to RSWT but with the energy flux density of the radial shock waves reduced to almost zero). Of the patients, 56.2% (9/16) that were treated with RSWT and 44.4% (4/9) of the patients treated with placebo reported (compared to baseline) a reduction in the VAS score greater than 50%, 6 months after the first session (defined as treatment success by the authors). This difference was not statistically significant (*p* > 0.05). However, the total mean VAS score of the patients treated with RSWT was reduced by 54.1% at 6-month follow-up but the total mean VAS score of the patients treated with placebo only by 3.9%. Marks et al. [9] concluded that there appeared to be a profound placebo effect in patients with heel pain, as well as a lack of evidence for the efficacy of RSWT in treating PF compared to sham therapy.

The paper by Marks et al. [9] has some weaknesses: (1) in the main text, the authors described an average duration of heel pain of 28.3 months before RSWT or sham treatment. On the other hand, the duration of symptoms was reported in Table 1 of the same paper as follows: 35.6 ± 43.2 days (mean ± standard deviation) (range, 1 to 180 days) for the patients treated with RSWT and 21.0 ± 16.4 days (range, 1 to 60 days) for the patients treated with placebo. As was true in the trial by Buchbinder et al. [29], Marks et al. [9] investigated mixed groups of patients suffering from either acute or chronic PF. Since more than 80% of PF patients experience resolution within 12 months, regardless of management [1], the approach by Marks et al. [9] could be considered an inadequate selection of PF patients for RSWT rather than reflecting inefficacy of RSWT treatment for this disease (similar to the study by Buchbinder et al. [29] discussed above). (2) This is further corroborated by the notion that at least one patient treated with placebo had a VAS score of 6 (with a maximum VAS score of 100) in the study by Marks et al. [9], which would translate into a VAS score of 0.6 in the studies by Chow and Cheing [7], Gerdesmeyer et al. [8], and Ibrahim et al. [11]. It remains unknown why such almost pain-free patients were enrolled in the study by Marks et al. [9].

Greve et al. [10] reported the results of 16 patients with chronic PF treated with RSWT (three sessions each 7 days apart, 2,000 impulses per session; EFD = 0.14 mJ/mm^2^, six impulses per second, group A), and another *n* = 16 patients with chronic PF to conventional physiotherapy (ten sessions of ultrasound, two sessions per week, plus exercises, group B). The authors found that both treatments were effective for pain reduction and improving the functional abilities of patients with PF (treatment success was not calculated as in the studies by Gerdesmeyer et al. [8], Marks et al. [9], and Ibrahim et al. [11]). However, the authors noted that the effects of RSWT occurred sooner than physiotherapy after the onset of treatment.

In summary, the lack of treatment success in the study by Marks et al. [9] using RSWT for PF can be explained by serious methodological shortcomings in this study (as in the case of the studies by Haake et al. [26] and Buchbinder et al. [29] on focused ESWT for chronic PF discussed above). In contrast, the studies by Chow and Cheing [7], Gerdesmeyer et al. [8], Greve et al. [10], and Ibrahim et al. [11] demonstrated that chronic PF can be treated successfully with RSWT. Most importantly, RSWT for chronic PF was demonstrated to result in long-term treatment success, demonstrated at both 6 [11] and 12 months [8] after the first treatment. These results justify the general recommendation to offer RSWT to patients 18 years of age or older with symptoms of PF for 6 months or more and a history of unsuccessful conservative therapy, before considering any surgical treatment.

### ESWT/RSWT vs. surgery in the treatment of chronic plantar fasciopathy

ESWT and RSWT have several advantages over surgery in the treatment for chronic PF, including minimally invasive percutaneous radio frequency nerve ablation propagated recently [38-42]. Because RSWT does not require local anesthesia, the procedure is completely non-invasive. In contrast, surgery has risks such as transient swelling of the heel pad, injury of the posterior tibial nerve or its branches, and flattening of the longitudinal arch with resultant midtarsal pain. In contrast to surgery, either open or endoscopic, ESWT and RSWT do not require patients to avoid weight bearing or a prolonged time for return to work. Rather, ESWT and RSWT allow patients to return to activities of daily life within 1 or 2 days, with an immediate return to most jobs and normal daily shoe wear.

## Conclusions

Both ESWT with focused shock waves and second generation RSWT are safe, effective, and easy treatments for chronic PF not responding to conservative therapy. Efficacy and safety of both ESWT and RSWT for chronic PF have been demonstrated in several RCTs in the international peer-reviewed literature. The lack of treatment success in some published RCTs using ESWT or RSWT can be explained by serious methodological shortcomings in the corresponding studies rather than reflecting inefficacy of ESWT or RSWT for this disease. Unlike surgery, ESWT and RSWT are non-invasive and can be performed as in office procedures, without the use of anesthesia. Furthermore, ESWT and RSWT do not require patients to avoid weight bearing or a prolonged time for return to work. The main advantages of RSWT over first generation focused ESWT are the lack of need for any anesthesia during the treatment and the demonstrated long-term treatment success (demonstrated at both 6 and 12 months after the first treatment using RSWT, compared to follow-up intervals of no more than 12 weeks after the first treatment using ESWT).

## Appendix

This appendix addresses the question whether the pressure waves generated with the Ossatron, Epos Ultra, Sonocur Basic, Orbasone, and Orthospec devices fulfill the characteristics set out by the following physical definition of shock waves [15,16]: (1) a high positive peak pressure (P_+_), sometimes more than 100 MPa but more often approximately 50 to 80 MPa, (2) a fast initial rise in pressure (T_r_) during a period of less than 10 ns, (3) a low tensile amplitude (P_−_, up to 10 MPa), (4) a short life cycle (I) of approximately 10 μs, and (5) a broad frequency spectrum, typically in the range of 16 Hz to 20 MHz. This question can be answered as follows: (1) the rise times of the pressure waves generated with the Sonocur Basic and Orbasone devices have not been published. Accordingly, it cannot be decided whether the pressure waves generated with these devices fulfill the characteristics set out by the physical definition of shock waves provided above. (2) The pressure waves generated with the Ossatron device were reported to have a rise time of 38 ns and a maximum peak positive pressure of 37.7 MPa [22,23]. Chitnis and Cleveland [22] characterized these pressure waves as shock waves. (3) The pressure waves generated with the Orthospec device were reported to have a rise time of 400 ± 100 ns and a maximum peak positive pressure of 34 ± 13 MPa [43]. Accordingly, the pressure waves generated with the Orthospec device are not shock waves according to the physical definition provided above. (4) Haake et al. [26] and Buchbinder et al. [29] treated patients suffering from PF with pressure waves generated with the Epos Ultra device. Buchbinder et al. [29] used various energy settings, i.e., within levels 1 to 9 (treatment began on level 1 and was gradually increased through to the highest tolerable level of pain for each participant). In contrast, Haake et al. [26] applied pressure waves with an EFD of 0.08 mJ/mm^2^ (i.e., level 3). The rise time of the pressure waves generated with the Epos Ultra device is approximately 600 ns at level 1, approximately 500 ns at level 3, approximately 200 ns at level 7, and less than 100 ns at level 9 (EMS Electro Medical Systems, Nyon, Switzerland; unpublished data). Furthermore, the positive peak pressure of the pressure waves generated with the Epos Ultra device is less than 7.5 MPa at level 1, less than 20 MPa at level 3, approximately 36 MPa at level 7, and approximately 56 MPa at level 9 (EMS Electro Medical Systems, Nyon, Switzerland; unpublished data). Accordingly, the pressure waves generated with the Epos Ultra device applied by Haake et al. [26] were not shock waves according to the physical definition provided above. The same holds true for the pressure waves generated with the Epos Ultra device at levels 1 to 7 applied by Buchbinder et al. [29]. This is in line with Cleveland et al.’s [23] notion that for treatment protocols at low energy settings, electromagnetic ESWT devices (such as the Epos Ultra) will not produce shock waves.

Accordingly, the pressure waves generated with the Orthospec device, as well as the Epos Ultra device operated at levels 1 to 7, do not fulfill the characteristics set out by the physical definition of shock waves provided above. Nevertheless, the pressure waves generated with these devices are named shock waves in the international peer-reviewed literature [26,28,29]. The same holds true for the pressure waves generated with the Ossatron device [22]. This indicates that in the field of musculoskeletal applications of pressure waves, several definitions of shock waves are used, irrespective of the type of generation or focusing of these pressure waves. This is also illustrated by the following characterization of shock waves applied to the musculoskeletal system by Rompe et al. [5]: (1) rise time < 1 μs and (2) positive peak pressure between 10 and 100 MPa (note that the pressure waves generated with the Swiss DolorClast have a positive peak pressure of more than 10 MPa when measured at 1 mm distance to the applicator. Chitnis and Cleveland [22] and Cleveland et al. [23] performed their measurements at 10 mm distance to the applicator).

Thus, the difference between the Ossatron, Epos Ultra, Sonocur Basic, Orbasone, and Orthospec devices on one hand, and the Swiss DolorClast, on the other hand, is not that the former devices produce shock waves and the latter one does not. Rather, the former devices generate focused pressure waves, whereas the latter one produces unfocused pressure waves, and both are named shock waves in the international peer-reviewed literature (Table 2).

**Table 2:** Characteristics of the pressure waves generated with various ESWT/RSWT devices marketed in the United States

**Wave characteristics**	**Devices generating focused shock waves**	**Devices generating radial shock waves**
Pressure waves that fulfill the characteristics set out by the physical definition of shock waves below^a^	Ossatron (SONOCUR Basic)^b^ and (Orbasone)^b^	
Pressure waves that do not fulfil the characteristics set out by the physical definition of shock waves below^a^	Orthospec and Epos Ultra^c^	Swiss DolorClast

The names of the corresponding manufacturers are provided in the main text. ^a^Physical definition of shock waves [15,16]: (1) a high positive peak pressure (P_+_), sometimes more than 100 MPa but more often approximately 50 to 80 MPa, (2) a fast initial rise in pressure (T_r_) during a period of less than 10 ns, (3) a low tensile amplitude (P_−_, up to 10 MPa), (4) a short life cycle (I) of approximately 10 μs, and (5) a broad frequency spectrum, typically in the range of 16 Hz to 20 MHz; ^b^rise time not published; ^c^when operated at levels 1 to 7 (i.e., as in clinical use for treatment of PF [26,29]).

It is important to note that the usability of shock waves for the treatment of the musculoskeletal system does not depend on whether these waves are shock waves according to the physical definition provided above or not. Rather, a significant tissue effect of these shock waves is cavitation consequent to the negative phase of the wave propagation [15]. Specifically, Schelling et al. [44] demonstrated stimulation of nerves with shock waves indirectly, i.e., via a cavitation-mediated mechanism. Nerve stimulation is nowadays hypothesized being a central mechanism of action of ESWT/RSWT for the musculoskeletal system [33-35]. As shown by Chitnis and Cleveland [22], both the Ossatron device (generating focused shock waves that fulfill the characteristics set out by the physical definition of shock waves provided above) and the Swiss DolorClast (generating radial shock waves that do not fulfill the characteristics set out by the physical definition of shock waves provided above) can induce cavitation (see also Figure 2).

## Competing interests

CS serves as paid consultant for and receives benefits from Electro Medical Systems S.A. (Nyon, Switzerland), the manufacturer and distributor of the Swiss DolorClast radial shock wave device. Accordingly, CS has received benefits for personal use from a commercial party related directly or indirectly to the subject of this article. However, CS, NC, JDR, HC, and JF declare that they have not received any honoraria or consultancy fee in writing this manuscript. No benefit was received or will be received directly or indirectly from a commercial party related to the performance of this study.

## Authors’ contributions

CS, NC, JDR, HC, and JF drafted the protocol of this study, searched references, collected data, performed data analysis, and helped in drafting the manuscript. All authors read and approved the final manuscript.
